# International Survey of the Tools Used for Assessment, Monitoring and Management of Home Mechanical Ventilation Patients

**DOI:** 10.3390/jcm12216803

**Published:** 2023-10-27

**Authors:** Michelle Chatwin, Nicholas Hart

**Affiliations:** 1 Royal Brompton Hospital, Guy’s and St Thomas’ NHS Foundation Trust, London SW3 6NP, UK; 2 National Hospital for Neurology and Neurosurgery, University College London, London WC1N 3BG, UK; 3 Lane Fox Respiratory Service St Thomas’ Hospital, Guy’s and St Thomas’ NHS Foundation Trust, London SE1 7EH, UK; nicholas.hart@gstt.nhs.uk; 4 Lane Fox Clinical Respiratory Physiology Research Unit, Centre for Human and Applied Physiological Science, King’s College London, London SE1 7EH, UK

**Keywords:** NIV, PVA, neuromuscular disease, chronic obstructive pulmonary disease, COPD, ventilatory failure, apnea hypopnea index, masks, leak

## Abstract

Background: There are limited data reporting diagnostic practices, compared to clinical guidelines, for patients with chronic respiratory failure requiring home mechanical ventilation (HMV). There are no data detailing the current use of downloaded physiological monitoring data in day-to-day clinical practice during initiation and follow up of patients on HMV. This survey reports clinicians’ practices, with a specific focus on the clinical approaches employed to assess, monitor and manage HMV patients. Methods: A web-based international survey was open between 1 January and 31 March 2023. Results: In total, 114 clinicians responded; 84% of the clinicians downloaded the internal physiological ventilator data when initiating and maintaining HMV patients, and 99% of the clinicians followed up with patients within 3 months. Adherence, leak and the apnea-hypopnea index were the three highest rated items. Oxygen saturation was used to support a diagnosis of nocturnal hypoventilation and was preferred over measurements of carbon dioxide. Furthermore, 78% of the clinicians reviewed data for the assessment of patient ventilator asynchrony (PVA), although the confidence reported in identifying certain PVAs was reported as unconfident or extremely unconfident. Conclusions: This survey confirmed that clinical practice varies and often does not follow the current guidelines. Despite PVA being of clinical interest, its clinical relevance was not clear, and further research, education and training are required to improve clinical confidence.

## 1. Introduction

Home mechanical ventilation (HMV) is a complex intervention that improves gas exchange, symptoms and health-related quality of life (HRQOL), decreases hospital admissions and increases survival in patients with chronic respiratory failure [1]. With the provision of HMV becoming more widespread, surveys of HMV delivery are important for informing clinicians, patients, healthcare policy makers and commissioners of care, and for providing data on the knowledge gaps that will drive the research, education and training agenda for clinicians. Over 20 years ago, the EUROVENT study reported the prevalence of HMV across Europe (6.6 per 100,000) and categorized the HMV users by the disease (lung/airways, thoracic cage and neuromuscular) causing their chronic respiratory failure, the interface used and the mode of ventilation [1]. More recently, the REINVENT study used a similar methodology and surveyed European HMV practice, but focused on patients with restrictive lung disease [2], and reported that amyotrophic lateral sclerosis was the major driver of HMV prescription. Furthermore, tests employed as part of the follow up included full polysomnography, portable sleep study, pulse oximetry (SpO_2_) and transcutaneous carbon dioxide (TcCO_2_) monitoring. However, there were no details provided regarding the diagnostic tests and monitoring of these patients.

The American Academy of Sleep Medicine (AASM) states that the gold standard method for documenting hypoventilation is the processing of an arterial blood gas (ABG) sample for determination of the arterial partial pressure of carbon dioxide (PaCO_2_) [3]. But they highlight that there can be difficulties in obtaining a direct measurement of PaCO_2_, and surrogate measures such as end-tidal CO_2_ (EtCO_2_) or TcCO_2_ are commonly used during polysomnography (PSG). Others consider diurnal PaCO_2_ as the gold standard for detecting hypoventilation, resulting in the indication for HMV, especially in neuromuscular disease (NMD) [4]. The AASM define the definition for nocturnal hypoventilation in adults as an increase in the arterial PaCO_2_ (or surrogate) to a value >55 mmHg for ≥10 min or a ≥10 mmHg increase in PaCO_2_ (or surrogate) during sleep (in comparison to an awake supine value) to a value exceeding 50 mmHg for ≥10 min [5]. Of course, these can only be obtained through overnight monitoring. When carrying out CO_2_ monitoring, the AASM recommend TcCO_2_ for titration studies [5]. 

Once established on HMV, the titration of HMV in patients with chronic respiratory failure should target a reduction in daytime PaCO_2_ [6,7,8,9] and, therefore, a post initiation overnight assessment of both SpO_2_ and TcCO_2_ and daytime PaCO_2_ are expected. Despite this recommendation, one HMV survey highlighted that clinicians expressed a low level of agreement with the investigations that are required prior to HMV prescription, with SpO_2_ as the main driver for the initiation of HMV in chronic obstructive pulmonary disease (COPD) [10]. In addition to this, the Swiss Pulmonary Society, who published the most recent guidelines to date, highlighted that CO_2_ monitoring, where available, should be carried out, and recommended follow up every 3 to 12 month following the initiation of NIV [7]. This guideline also recommended device data interrogation to assess ventilator adherence. In addition to a target adherence of greater than 4 h per night, the guideline indicated a requirement for the detection and correction of mask leaks, as this is a major cause of patient-ventilation asynchrony (PVA). PVA has been reported to reduce adherence [11] and survival [12] from observational studies; however, clinical study data have shown that PVA does not impact overnight gas exchange [13]. Although the clinical importance of PVA has yet to be determined in a clinical trial, the assessment, monitoring and management of PVA have been reported. Despite these recommendations, the rate of adoption of PVA assessment in routine clinical practice is unknown. 

This comprehensive survey was designed to evaluate the physiological and ventilator download tools that are employed to diagnose nocturnal hypoventilation, as well as the timing and tools used by HMV clinicians in patient follow up in order to determine the clinical response. In addition, this survey sought to evaluate the clinical importance of each of these physiological and ventilator download tools. Finally, with the growing interest in the area, the survey evaluated the requirement for, and use of, PVA assessment in clinical practice and determined the confidence of the respondents in identifying PVA.

## 2. Materials and Methods

We designed an anonymous (GDPR compliant) survey that aimed to register the current practice of monitoring patients initiated on non-invasive ventilation (NIV). The survey was open between 1 January 2023 and 31 March 2023. Section 1 explored the characteristics and experience of the responder and the size and experience of their center. Section 2 evaluated their current assessment and monitoring practice, including a rating of clinical importance. Section 3 focused on PVA; in particular, the use of PVA assessment and management in their day-to-day clinical practice, their knowledge of the previous SomnoNIV publications [14,15] and barriers to assessment, monitoring and management of PVA, as well as the educational tools required to enhance confidence. Prior to posting the questionnaire online, it was trialed for usability and comprehension with 6 clinicians (2 pulmonologists, 3 physiotherapists, 1 technician) and modified for English language and content prior to sending out, with the recommended changes inputted. The full questionnaire is available online: https://docs.google.com/forms/d/e/1FAIpQLScIdbhTSe_Aae-iKm7Ixy7TgjMAa8jeubvp6282mN8N_ukVmQ/viewform?usp=pp_url (accessed on 27 September 2023).

## 3. Results

### 3.1. Country of HMV Delivery 

**Figure 1:**
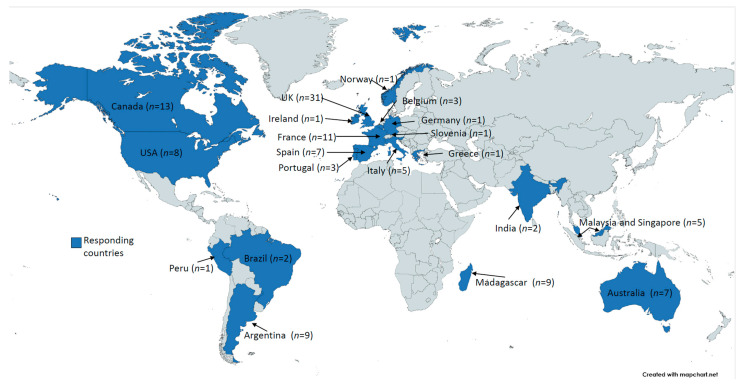
This figure shows the countries of respondents to the survey in blue, along with the number of respondents per country. Abbreviations: United Kingdom (UK), United States of America (USA).

We received 114 responses from around the world (Figure 1). For the purpose of analysis, the respondents were split into three regions: North America (*n* = 21), Europe (*n* = 67) and the rest of the world (ROW) (*n* = 28). The United Kingdom provided the greatest number of respondents, with 31 respondents.

### 3.2. Type of HMV Center

Overall, 56.9% of the respondents were from university hospitals. Table 1 shows the respondents’ institutions, and Table 2 shows the professional background of the clinicians (61.0% were medical doctors).

**Figure 2:**
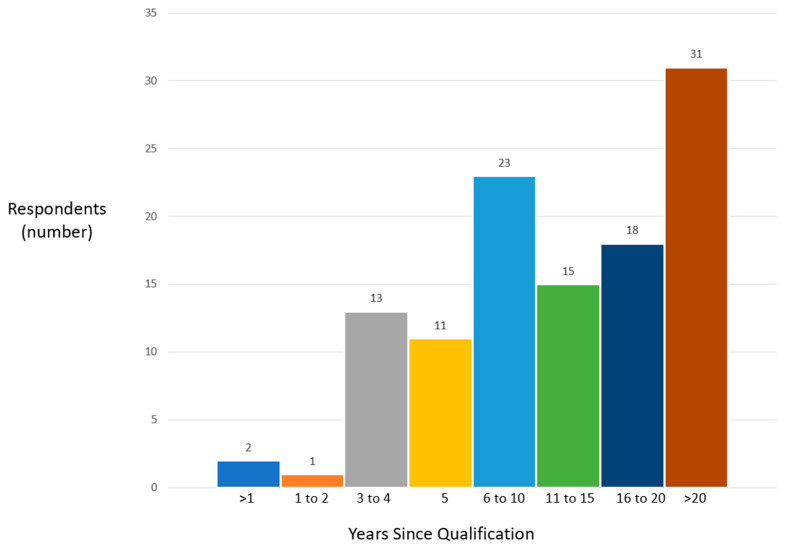
This figure shows the number of years since qualification of the respondents.

Sixty-nine percent of respondents had been qualified for more than 5 years; a breakdown of the years since qualification is shown in Figure 2.

### 3.3. Mode of HMV Treatment

**Figure 3:**
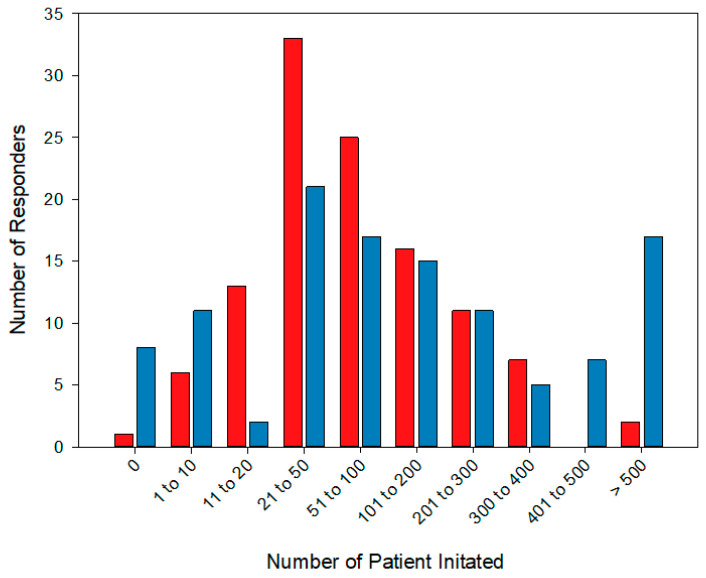
This figure shows the number of patients set up on noninvasive ventilation (NIV) (red bars) and continuous positive airway pressure (CPAP) (blue bars) per year by responder.

Eighty-two respondents (76.3%) reported that they initiated both noninvasive ventilation (NIV) and continuous positive airway pressure (CPAP), while twenty-five (21.9%) only initiated NIV and two (1.8%) only initiated CPAP. There was no country that initiated only NIV or only CPAP. Figure 3 highlights the number of patients that received CPAP or NIV in the categories defined in the survey. Most respondents initiated between 20 and 200 patient set ups on HMV per year.

### 3.4. Pediatric, Transition and Adult Service

Out of the 114 respondents, 46% (*n* = 52) treated only adult patients, 34% (*n* = 39) respondents treated adult and transition patients, while 11% (*n* = 12) treated only pediatrics, 5% (*n* = 6) treated adult, transition and pediatric patients and 4% (*n* = 5) treated both adult and pediatrics; no respondents only treated transition patients. There was no single profession more likely to treat an adult and pediatric patient, and there was no difference between the geographical regions.

### 3.5. HMV Treatment by Diagnosis

NIV treatment by diagnosis demonstrated 96% (109/114) of the respondents’ treated neuromuscular disorders (NMD), 93% (106/114) obesity hypoventilation syndrome, 89% (101/114) chest wall disease, 84% (96/114) chronic obstructive pulmonary disease (COPD), 84% (50/114) bronchiectasis, 74% (84/114) central hypoventilation syndrome, 70% (80/114) spinal cord injury, 65% (74/114) treated obstructive sleep apnea patients (OSA), 42% (48/114) cystic fibrosis, 33% (38/114) heart failure and 25% (29/114) treated other suppurative lung disease patients.

### 3.6. HMV Set Up

**Figure 4:**
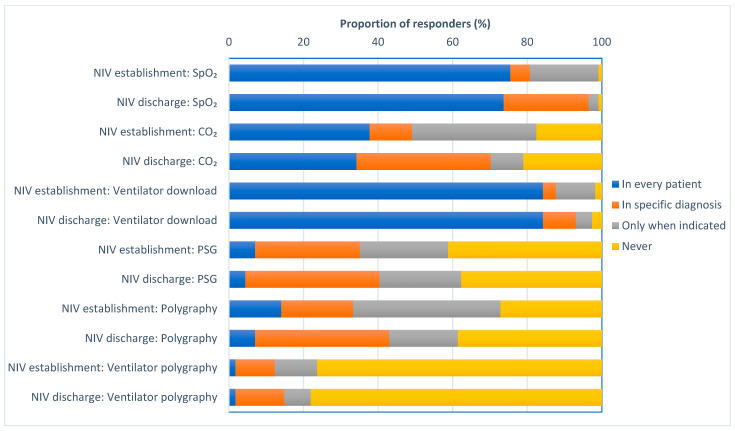
This figure shows the proportion of respondents who performed each type of monitoring at HMV initiation and at discharge. Respondents stated if they would perform this type of monitoring in every patient (blue), in specific diagnosis (orange), only when indicated (light grey) or never (yellow).

In addition, 114 people responded to the HMV set up questions. Data downloaded from the in-built ventilator software were used for the majority of patients when establishing NIV and when established on NIV prior to discharge. SpO_2_ and CO_2_ monitoring were the most common types of monitoring undertaken (see Figure 4). The respondents in Spain, Italy and Belgium utilized SpO_2_ monitoring when establishing patients on HMV. All respondents in the USA and most from Italy, Spain and the UK downloaded data from the in-built ventilator software when initiating HMV. Routine full polysomnography was used infrequently. After establishing HMV, a lower number of respondents used CO_2_ monitoring to evaluate the efficacy of HMV. Only Australian respondents reported using the downloaded ventilator data for all patients at discharge.

### 3.7. Post Discharge HMV Follow Up

Overall, 112 clinicians responded to how they would follow up patients after discharge; 88% (*n* = 98) responded that they would book a clinic appointment, 65% (*n* = 73) download the ventilator data as part of the follow up, 64% (*n* = 72) use telemonitoring and 48% (*n* = 54) offer telephone consultations, 20% (*n* = 22) would admit their patients to hospital for clinical review, with 30% (*n* = 34) of respondents admitting their patients for a sleep study. Home visits were used only for follow up by 2.7% (*n* = 3), and 0.9% (*n* =1) did not follow up their patients. Clinicians could choose more than one option for the follow up of their patients, indicating that follow up is often based on the clinical need of the patient. There was no difference between countries or geographical regions.

With regards to the timing of the follow up of newly initiated patients, 99% (*n* = 109) follow up their patients within the first 3 months. Specifically, 32% (*n* = 35) patients were followed up at 2 weeks, 32% (*n* = 35) at 4 weeks, 12% (*n* = 13) at 6 weeks, 8% (*n* = 9) at 8 weeks, 16% (*n* = 17) at 12 weeks and 1% (*n* = 1) at 6 months.

### 3.8. HMV Monitoring

**Figure 5:**
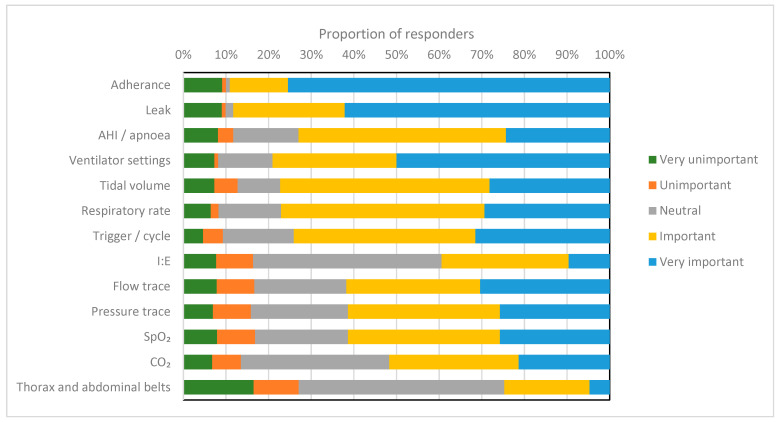
This figure shows the proportion of responders (%) rating for importance with regards to the options on the vertical axis. Respondents were asked to classify what was very unimportant (green), unimportant (orange), neutral (grey), important (yellow) and very important (blue). Apnea hypopnea index (AHI), inspiratory: expiratory ratio (I:E), oxygen saturation (SpO_2_) and carbon dioxide (CO_2_).

In total, 110 clinicians (98%) responded that they reviewed the downloadable ventilator data. Among them, 86 (78%) of the respondents report that this is performed in all patients, while 24 (22%) apply this only to selected patients. Among the respondents who reviewed the downloaded data, 100% (*n* = 111) evaluated the adherence data, with 99% (*n* = 109) reviewing the leak data. Furthermore, 90% (*n* = 100) reviewed the apnea hypopnea index (AHI) or apnea index (AI). Ventilator settings (inspiratory and expiratory positive airways pressures) were reviewed by 95% (*n* = 105), with the tidal volume being reviewed by 87% (*n* = 97) and respiratory rate by 86% (*n* = 95). Cycling and triggering were reviewed by 84% (*n* = 93) and the I:E ratio by 54% (*n* = 60) of the respondents. Flow and pressure traces were reviewed by 53% (*n* = 59) and 52% (*n* = 58) of the respondents, respectively. SpO_2_ (41% (*n* = 45)), CO_2_ (16% (*n* = 18)) and thoracic and abdominal belts (8% (*n* = 9)) were the least reviewed. Adherence followed by an unintentional leak was reported as the most important factor to review from the ventilator download data. All of the items that were rated are reported in Figure 5. 

### 3.9. In-Built Ventilator Software Data Download

Overall, 96% (*n* = 105) of the respondents review the leak data, 85% (*n* = 93) for residual obstructive events, 81% (*n* = 88) for pressure support delivery, 79% (*n* = 86) for percentage of triggered breaths and 58% (*n* = 63) for decreased respiratory drive. Again, the clinicians could provide multiple responses.

**Figure 6:**
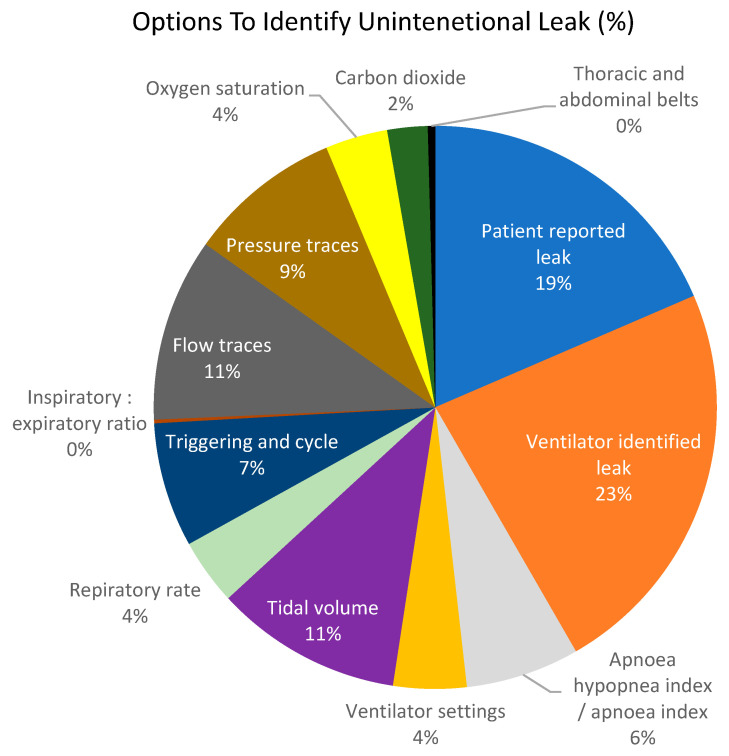
This figure shows the proportion of responders (%) who would use each option to identify an unintentional leak.

The responders were given various parameter choices and were asked how they identify leaks. In total, 110 clinicians (97%) responded to this question and were able to respond to more than one option if they used more than one item to identify a leak. Figure 6 shows the percentage of responses for each option. Interestingly, more respondents would pay attention to a leak reported from the ventilator over the patient reporting a leak.

We asked the respondents about the tools they use to help differentiate between central and obstructive events. Overall, 108 responded (95%); 57% (*n* = 61) used the downloadable ventilator data, 55% (*n* = 59) used respiratory polygraphy, 25% (*n* = 27) used SpO_2_ and 16% (*n* = 17) used thoracic and abdominal belts that can be integrated into the ventilator. There were no respondents using thoracic and abdominal belts from North America.

In total, 110 clinicians responded to the question focused on identifying nocturnal hypoventilation, with responders able to give multiple answers. Among them, 72% (*n* = 79) used SpO_2_, 66% (*n* = 73) used TcCO_2_, 61% (*n* = 67) used arterial blood gas (ABG) measurement, 18% (*n* = 20) used EtCO_2_, 15% (*n* = 16) used venous blood gas (VBG) and 23% (*n* = 25) used other methods. Fifty-five percent (*n* = 43) of the respondents indicated that they performed an ABG to confirm the end tidal or TcCO_2_ measurement. To identify nocturnal hypoventilation in a patient on HMV, 68% (*n* = 73) used TcCO_2_, 62% (*n* = 67) used ABG, 14% (*n* = 15) used EtCO_2_ and 11% (*n* = 12) used VBG measurement. Respondents in European countries did not report the use of EtCO_2_.

### 3.10. Patient Ventilator Asynchrony

Seventy-eight percent (*n* = 87) of clinicians responded that they would review PVA. The respondents who do not review PVA were asked to identify what they considered to be the barriers from a list. These options included: lack of equipment (44%, *n* = 15), lack of confidence in interpreting PVA (41%, *n* = 14), ventilators lack capacity to download the data (41%, *n* = 14), lack of education and support in this area (38%, *n* = 13), cost (6%, *n* = 2), unclear of the significance (3%, *n* = 1), time consuming (3%, *n* = 1) and no clinical requirement (3%, *n* = 1).

Eighty-eight (77%) of the clinicians responded regarding the tools they used to identify PVA. The tools used included ventilator flow and pressure curves (78%, *n* = 69), respiratory polygraphy (26%, *n* = 23), a combination of the flow, pressure curves, respiratory polygraphy (41%, *n* = 36), ventilator integrated polygraphy (11%, *n* = 10), SpO_2_ (40%, *n* = 35) and TcCO_2_ (24%, *n* = 21). Others reported utilizing a day session in the sleep laboratory, auscultation, esophageal monitoring and electromyography (EMG), but in each case, this was only one respondent.

Eighty-nine (78%) responded detailing the reasons for reviewing PVA; these included optimizing ventilator settings (92%, *n* = 82), increasing adherence (88%, *n* = 78), improving patient comfort (88%, *n* = 78), correcting a leak (45%, *n* = 40), improving survival (37%, *n* = 33), academic or research interest (6%, *n* = 5), improving sleep quality (1%, *n* = 1) and decreasing symptoms (1%, *n* = 1).

**Figure 7:**
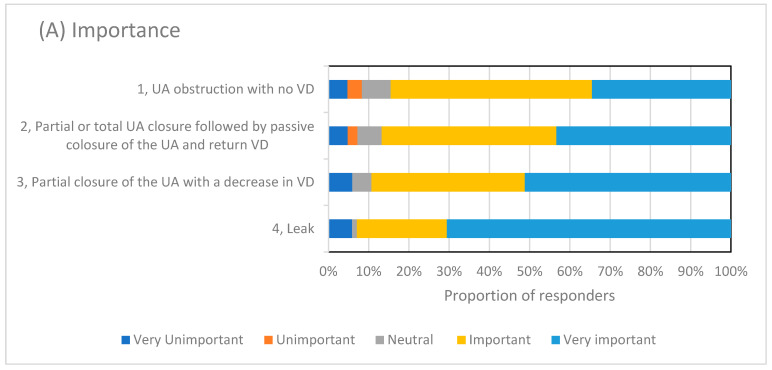

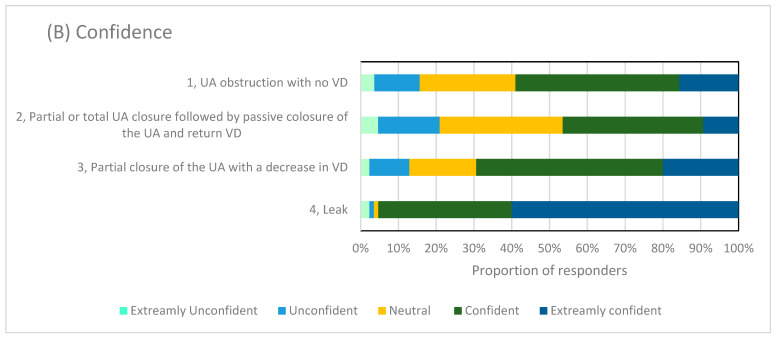
Shows the proportion of responders’ (%) views on the four types of patient ventilator asynchrony defined by Gonzalez and co-workers [14]. (**A**) shows the proportion of respondents who identified each PVA with regards to their importance. (**B**) shows the ratings for confidence of the respondents in identifying each PVA. Upper airway (UA), ventilatory drive (VD).

When asked specifically about the systematic analysis of polygraphy (PG) or polysomnography (PSG) for identifying and scoring abnormal events occurring during NIV [14], 47 out of the 91 (52%) respondents were aware of the published data. The clinicians were then asked to rate events in terms of importance and their confidence in identifying the four events described in the proposed systematic analysis [14]; e.g., partial or total upper airway obstruction without a reduction in ventilatory drive; partial or total upper airway obstruction with a reduction in ventilatory drive followed by passive closure of the upper airway and resumption of respiratory drive; partial or total upper airway obstruction with a reduction in ventilatory drive; and a leak. Most respondents rated all of the events as important or very important, with leak identification being rated as the most important event and most respondents feeling very confident in identifying it. All of the results are shown in Figure 7.

**Figure 8:**
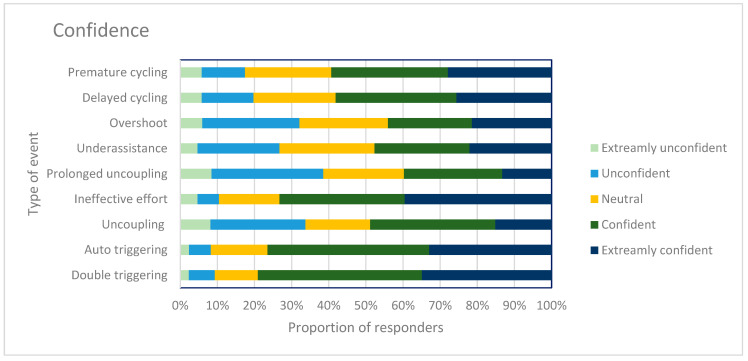
This figure shows the proportion of responders’ (%) confidence when identifying a further nine types of asynchronies identified in “Framework for patient-ventilator asynchrony during long-term non-invasive ventilation” [15]. The darker the color, the higher the level of confidence.

When asked if they had read the “Framework for patient-ventilator asynchrony during long-term non-invasive ventilation” [15], 53 (58%) responded that they had read the framework. The algorithm from the paper was shown in the questionnaire. In total, 81% (*n* = 66) found it a useful tool. This paper highlights and describes further PVAs that clinicians were asked to rate their confidence in identifying. The PVAs were premature cycling, delayed cycling, overshoot, under assistance, prolonged uncoupling, ineffective effort, uncoupling, auto triggering and double triggering. Clinicians were far more confident in identifying auto triggering, double triggering and ineffective effort (see Figure 8). A lack of confidence was not related to either less years of practice in the area or to profession.

**Figure 9:**
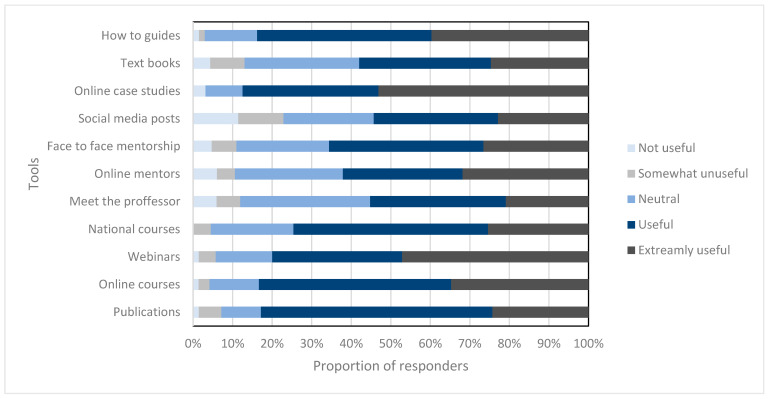
This figure shows the proportion of responders’ (%) ratings of usefulness for various methods of teaching, which are listed in the Y axis. The darker the color, the more useful the option.

When the clinicians were asked what tools would help to increase their confidence in identifying PVA, the responses included: online publications in this area, online intensive courses, webinars, local face to face courses, national face to face courses, meet the professor sessions, online mentorship, face to face mentorship, social media posts, regular online case studies, a textbook and how to identify PVA online guides. When asked to rate what would be useful to improve their knowledge and confidence, online case studies was deemed to be the best tool; however, more publications in the area are clearly warranted. The ratings for all of the options are shown in Figure 9.

## 4. Discussion

This is the first comprehensive survey to evaluate the clinical practice for the assessment, monitoring and management of HMV and extends the data from the previous studies of diagnosis, interfaces, modes and HMV settings [1,2,10]. The majority of the clinicians reported using the downloaded physiological and ventilator data when initiating and maintaining HMV patients, with the majority of patients being followed up within 3 months. Adherence, leak and the apnea-hypopnea index were the three highest rated assessment and monitoring tools. Interestingly, the use of oxygen saturation to support a diagnosis of nocturnal hypoventilation was preferred over a measurement of carbon dioxide, with over three-quarters of the clinicians reviewing the data for PVA assessment, although the confidence in identifying PVAs was low to moderate.

### 4.1. Limitations of Study

Unlike previous surveys [1,2,10], this was an open survey that was advertised through social media platforms and scientific societies; therefore, this survey lacks a denominator, and a response rate cannot be reported. The survey may not represent the average behavior of all the specialists involved in HMV as the majority of the 114 clinicians that responded had more than 5 years of experience and were from university or large hospitals. Thus, the responses likely represent the most advanced models of care in highly specialized settings. Despite this, the data herein retain significant clinical importance. Indeed, the respondents reported an average HMV initiation per year of 105 patients, which further highlights the clinical experience and interest of the respondents. Like in the previous data, there were variations in the clinicians’ practice reported within the same country and across the world [10], although over 50% of the respondents were from Europe. From these data, there were no clear differences between Europe, North America and the rest of the world in terms of overall clinical practice. The fact that our survey highlights that there is a need for better education and training in this area emphasizes the importance of implementing standard educational programs on HMV.

### 4.2. Tools to Assess, Monitor and Manage Nocturnal Hypoventilation

Although within-country practice varies, patients with NMD are the largest group of patients initiated on HMV, followed by COPD and obesity-related respiratory failure, which is consistent with previous data [1]. Furthermore, in line with previous EUROVENT data [1], other chronic lung diseases are treated with HMV. Despite guidelines [3,5] stating that carbon dioxide measurements (arterial blood gas or transcutaneous) are mandated in order to make a diagnosis of nocturnal hypoventilation—with recent data highlighting a targeted reduction in daytime and nocturnal carbon dioxide levels improving the patients’ clinical outcome [6,7,16,17,18,19,20]—the current survey showed that when initiating HMV and prior to discharge post initiation, the SpO_2_ monitoring and physiological and ventilator downloaded data were employed more frequently than carbon dioxide measurements. Surprisingly, some respondents reported that they never use carbon dioxide measurements to titrate HMV. The current data support the previous data of Crimi et al., which demonstrated that SpO_2_ monitoring was the primary tool for HMV set up and initiation [10]. Greater clinical education and training in terms of the benefits of targeted carbon dioxide reduction are required and, importantly, the standardization of the assessment, monitoring and management of chronic respiratory failure must aim to target and reduce carbon dioxide. SpO_2_ should not be used alone like it is as a screening tool; targeted carbon dioxide reduction must be a priority [21]. We can only speculate on the reason for this unexpected finding; however, the location of HMV initiation (e.g., in the home or ambulatory outpatient setting) with a lack of access to arterial blood gas measurements and TcCO_2_ could be a factor. However, the responses received in the survey indicated that equipment ABGs and TcCO_2_ monitoring were available.

### 4.3. Physiological and Ventilator Download Data

Physiological and ventilator download data are now common practice; almost all of the respondents reported the use of these accessible data, with over three-quarters of the respondents applying them to all of their patients. The three most frequently reviewed parameters were ventilator adherence, mask-leak and the residual apnea or apnea hypopnea index, which supports the clinicians’ perception that these parameters are markers of HMV clinical efficacy supported by guideline evidence [21]. Specifically, there is limited clinical benefit with low ventilator adherence [22,23,24], significant mask leak [25,26] and a persistently elevated apnea hypopnea index and residual apnea [11]. Furthermore, in some countries, reimbursement is only provided if the patient adheres to the HMV therapy. A further unexpected finding was that SpO_2_ and CO_2_ monitoring were reviewed less frequently from the ventilator download data than adherence and the leak and apnea and apnea hypopnea index, although we acknowledge that external equipment is required for such monitoring and these parameters are not considered part of the ventilator download. Furthermore, SpO_2_ and CO_2_ monitoring tend to be used when there are clinical issues raised by the patient or observed from the downloaded data; e.g., poor adherence, high leak and residual apneas and persistent high apnea hypopnea index. One-hundred percent (*n* = 110) of the responders review the ventilator download data for unintentional leaks. It is surprising that clinicians rely more on the ventilator download data for unintentional leaks rather than directly questioning the patient during a clinical review (88%, *n* = 80). Finally, most of the respondents were neutral concerning the use of ventilator integrated polygraphy, which is likely due to the lack of data detailing its clinical role outside of case series [27].

### 4.4. Patient-Ventilator Interaction

Respiratory polygraphy (RPG) or polysomnography (PSG) with TcCO_2_ are considered the gold standard for documenting residual respiratory events occurring under HMV. However, PSG is not readily available. In the current survey, over 35% of the clinicians report that they would never use RPG or PSG in patients, despite being highlighted in expert consensus and guidelines [14,15,28], most likely because RPG or PSG requires physiological expertise and time to set up and score, which has driven the field toward physiological and ventilator download data. The survey identified that the use of in-built ventilator software is important to differentiate between central and obstructive events, despite the wide variation in the reported data accuracy [29]. 

This survey also evaluated the clinical utility of PVA. Of clinical importance, over three-quarters of the clinicians review the available data and undertake an assessment of PVA. We are, unfortunately, unable to report if this is diagnosis-specific, but the respondents highlighted a lack of equipment for assessing PVA and a lack of clinical confidence in interpreting the data. For those who reported assessing and reviewing PVA, most use the in-built ventilator software, despite the gold standards being RPG and PSG. The main drivers for assessing PVA were to optimize the ventilator settings and to increase adherence, rather than to correct an unintentional leak, which is the first PVA to be evaluated [14,15]. Indeed, without correcting the leak, it is not possible to accurately identify any other forms of PVA. Only sixty percent of the clinicians were aware of the SomnoNIV guidance [14,15], and when asked to rate the systematic analysis of four major types of PVA, a leak was identified as the most important and most of the respondents felt confident or very confident in identifying an unintentional leak.

From the current survey, the confidence in identifying the other three PVAs described by the SomnoNIV group [14] was dependent on the clinical importance of the event. The SomnoNIV group, in an extension of the framework for PVA, describe a total of 13 PVAs, which is similar to the PVAs reported by Ramsay et al., although this study employed parasternal electromyography (EMG) as a more detailed and accurate measure of neural drive to breath [13]. There was evident clinical confidence in identifying the common PVAs and those described widely in the intensive care setting–triggering asynchronies, e.g., ineffective effort, auto-triggering, double triggering, and cycling asynchronies, e.g., premature or delayed cycling. The clinical utility of PVA remains to be determined, as although observational retrospective studies have reported a relationship between PVA and ventilator adherence [11] and survival [12], there are detailed prospective physiological data demonstrating that PVA does not impact overnight gas exchange [13]. In addition to more research in this area, it would be useful to have a patient–ventilator interaction education and training program. From our survey, the tools that rated highly for confidence and usefulness included how to guides, online webinars and face to face courses. This survey can act as a basis to streamline and enhance the knowledge in the field of patient–ventilator interaction in HMV patients.

## 5. Conclusions

This is the first comprehensive survey to evaluate the clinical practice for the assessment, monitoring and management of HMV, and extends the data from previous studies. The survey may not represent the average behavior of all clinicians as the respondents were from university or large hospitals and likely represent the most advanced models of care in highly specialized settings. This highlights the importance of implementing standard educational programs on HMV. The majority of the clinicians reported using the downloaded physiological and ventilator data when initiating HMV patients, with the majority of patients being followed up within 3 months. Adherence, leak and the apnea-hypopnea index were the three highest rated assessment and monitoring tools. Interestingly, the use of oxygen saturation to support a diagnosis of nocturnal hypoventilation was preferred over a measurement of carbon dioxide, with over three-quarters of the clinicians reviewing the data for PVA assessment, although the confidence in identifying PVA was low-to-moderate.

## Figures and Tables

**Table 1:** Respondents’ Institution.

Type of Institution	Number
Tertiary care and above	4
University Hospital	68
General Hospital	26
Childrens Hospital	2
Private Hospital	5
Home care program	6
Sleep facility	2
Rehabilitation center	1

**Table 2:** Respondents’ profession.

Type of Institution	Number
Pulmonologist/Respiratory Physician	55
Intensivist	9
Pediatrician	5
Physiotherapist/Respiratory therapist	30
Nurse	11
Physiologist	4

## Data Availability

Data is contained within the article any further information can be requested from the authors. The survey is available online: https://docs.google.com/forms/d/e/1FAIpQLScIdbhTSe_Aae-iKm7Ixy7TgjMAa8jeubvp6282mN8N_ukVmQ/viewform?usp=pp_url (accessed on 26 September 2023).
